# Antecedents and consequences of relationship quality in pharmaceutical industries: A structural equation modelling approach

**DOI:** 10.1371/journal.pone.0279824

**Published:** 2023-01-20

**Authors:** Muhammad Turki Alshurideh, Barween Al Kurdi, Hamzeh Almomani, Zaid Mohammad Obeidat, Ra’ed Masa’deh

**Affiliations:** 1 Department of Marketing, School of Business, The University of Jordan, Amman, Jordan; 2 Department of Marketing, Faculty of Economics and Administrative Sciences, The Hashemite University, Zarqa, Jordan; 3 University of Reading, Reading, United Kingdom; 4 Department of Management Information Systems, School of Business, The University of Jordan, Amman, Jordan; Public Library of Science, UNITED KINGDOM

## Abstract

This study aims to investigate the antecedents and consequences of relationship quality in the Jordanian pharmaceutical industry. A convenience sampling technique was used to select a representative sample of physicians working in the public healthcare sector in Jordan. Particularly, 500 questionnaires were distributed and 374 questionnaires were used in the analyses. Structural Equation Modeling was used to test the research hypotheses. Results revealed that the relationship quality was affected positively by the following antecedent variables (relational selling behavior, expertise, and ethical Relationship) while similarities had no significant effect on the relationship quality. The findings also revealed that the anticipation of future interaction between the physicians and medical representatives was affected positively by relationship quality. This study is the first that adequately examined the relationship quality and the anticipation of future interaction in the Jordanian pharmaceutical sector.

## 1. Introduction

Relationship marketing is generally concerned with how organizations create, build and maintain productive relationships with customers for long-term profitability [1]. Besides, relationship marketing centers on all activities directed towards establishing, developing and maintaining successful exchange with customers and other constituents [2]. Servicing and selling to current customers is considered important for long-term marketing success and for winning new customers [3]. As a result of increased competition, relationship marketing has been responsible for changing and shifting business concepts from “winning new customers” to “caring for and keeping current customers” [4]. A firm that is considered successful in developing strong relationships with customers ensures an important and durable competitive advantage that is hard for competitors to understand, copy or displace [2].

One driver of relationship effectiveness and success relates to the concept of relationship quality [2, 5], which refers to the customer’s ability to rely and trust the salesperson’s or firm’s integrity owing to consistent and satisfactory previous interactions with them [6, 7]. This concept was found to be a strong predictor of any future interaction between the firm and its customers [7]. Within the B2B context, however, limited research attention has been given to examining the relationship quality within the pharmaceutical industry with most of the research undertaken to be conceptual [8].

In general, in a B2B setting, firms must understand the nature and conditions of their targeted clients because of their unique characteristics and consider that B2B customers purchase larger quantities and have more specialized needs [9]. Consequently, understanding these needs requires an understanding of what they deem as quality service for firms to retain them successfully. Furthermore, the retention of B2B customers is often essential and leads to greater revenues for firms [10]. Subsequently, considering that profitability often depends on consumer loyalty [11, 12], and loyalty depends on the quality of the service provided [2], understanding the antecedents and components of what constitutes a quality service within the B2B context is vital. Moreover, within the pharmaceutical context, understanding the essential dimensions of quality relationships will often lead to greater brand equity and value owing to the increased prescription behavior by physicians and sales representatives [8] and improve customer (i.e. physicians) loyalty [13]. Also, this will lead to better use of their marketing resources which could result in cost reductions and, thus, lowering the costs of products for consumers [14]. As a result, both physicians and researchers would benefit from understanding the antecedents and consequences of relationship marketing practices in the pharmaceutical industry.

In this paper, section one examines the previous literature on pharmaceutical marketing. Afterward, the study model, hypotheses development, and a review of related previous literature on the impact of relationship quality on customer loyalty are discussed. Section three presents the study’s methodology including the population and sample, measures, data collection instrument and response rate, validity constructs and composite reliability. The fourth section illustrates the empirical results, followed by the discussion. The final section includes the conclusion, study limitations, and recommendations.

## 2. Marketing in the pharmaceutical industry

The literature of pharmaceutical marketing has recently shifted its focus to examine the impact of social media on pharmaceutical firms [15–20]. Generally, this theme of research examined the current trends of social media marketing and the main ways social media could improve pharmaceutical marketing activities and suggested that using mobile apps, creating a digital marketing structure, establishing a strong IT support as well as partnerships with successful IT firms could increase the impact of pharmaceutical marketing activities [17, 19]. In addition, this trend of research identified that online customer engagement now is one of the best pharmaceutical relationship marketing tactics [18]. This trend also suggested that providing more product information using social media channels such as forms, blogs, Facebook, Twitter and creating a drug online resource library in addition to creating and using keywords will greatly enhance the effectiveness of pharmaceutical marketing [20]. Despite this current trend, the majority of research in the pharmaceutical marketing literature has focused on examining and identifying the factors affecting the prescription behavior of the physician [21–32]. Generally, several factors within the pharmaceutical marketing literature influence physician’s prescription behavior [33]. A variety of factors were used to investigate the influence of pharmaceutical marketing on prescription behavior like the doctor-based attributes, treatment costs, and patient preferences [34]. In addition, a strong emphasis was given to examining the influence of promotional factors on the physicians’ prescriptions including the pharmaceutical firm reputation, sales rep visits, new drugs, brochures, physician experience, word of mouth by peers, and training.

Overall, this trend identified factors affecting prescription behavior such as scientific information by public authorities [22], service quality [27], public relations and sales promotions [21], drug performance and expectations [26], social interactions and peers effect [24], detailing in salesforce effort [33], and marketing mix elements [25, 35]. Moreover, a number of studies within this stream of research focused on the on examining the influence detailing of medical representatives. This trend of research uncovered positive relationships between detailing frequency, detailing roles and policies and the decision to prescribe as well as the profile of the physician [26, 29, 33, 36, 37]. Additionally, the findings of this stream identified a significant relationship between time and physicians’ responsiveness to detailing [38]. Moreover, evidence also suggests that firms that have strong detailing practices are more hurt by governmental restriction policies [28, 39–42]. Another study by [31] identified two main roles for detailing (i.e. informative and persuasive) in which the informative role often relates to the chemical component aspect and the persuasive role often relates to the brand. [32] also found a positive relationship between detailing and written prescriptions.

While the majority of the literature focused on these two main research themes, limited research attention has been given to the antecedents and consequences of relationship quality and its impact on pharmaceutical firms. Pharmaceutical marketing varies from most customer marketing activities in many aspects. Aside from the heavy use of patent protection of products and the increasing incentives for research and development [8]. In the pharmaceutical industry, the physician often plays a bigger role in deciding what treatment the consumer (or patient) gets in the end. As a result, the physician is the main target of the pharmaceutical marketing efforts owing to his/her role as an intermediary between the firm and consumer [8]. Besides, most promotional activities for pharmaceutical firms such as advertising are prohibited in many countries, which necessitate the use of other promotional tools such as personal selling, which is a method that is highly dependent on establishing rapport and personal relationships with the consumer [40]. Consequently, the role of relationship marketing in the pharmaceutical industry and the B2B context is essential considering that marketers recognize that it costs less to keep current customers than to acquire new ones [43]. As a result, pharmaceutical firms build loyalty schemes to retain their current customers, especially physicians to influence their prescription behavior [30]. Consequently, as it is important to establish and retain good rapport with clients in the pharmaceutical industry, this study will examine not only the antecedents of establishing a quality relationship with clients in the pharmaceutical industry but also its outcomes.

## 3. Conceptual framework

### 3.1. Relationship quality

Relationship quality has a critically important role in relationship marketing and is mainly recognized as a central construct [7]. Even though there is no consensus on which dimensions make-up relationship quality [43–46], considerable overlap exists in its various conceptualizations. Many studies such as [5, 47, 48] replicated the relationship quality conceptualization adopted by [7] using the same dimensions, namely, trust and satisfaction. Several other studies such as [47–49] expanded this list by incorporating a new commitment dimension to relationship quality. Similarly, in the B2B context, customer satisfaction, trust and commitment are the essential components in relationship quality.

Trust refers to the “perceived credibility and benevolence of a target of trust” [50], and several previous pieces of literature agrees that trust is composed of two elements, namely, credibility and benevolence [51–55]. With trust in a partner’s credibility relating to the belief that one’s partner stands by his/her word, fulfills promised role obligations and is sincere. Trust in a partner’s benevolence relates to the belief that one’s partner is interested in the firm’s welfare and will not take unexpected actions that would harm the firm [52]. Satisfaction, on the other hand, is considered as a feeling reaction to the perceived difference between performance appraisal and expectations [56], and is often measured through two dimensions in the B2B context, firstly, an economic one that relates to the evaluation of the economic outcomes that flow from the relationship, and, secondly, a social one that relates to the psycho-social aspects of the relationship [57, 58].

Finally, relationship commitment is considered as an attitude, where the main assumption is that the relationship is worth the effort to be maintained [59]. [60] defines commitment as “an exchange partner believing that an ongoing relationship with another is so important as to warrant maximum efforts at maintaining it” (p. 23). [61] simplifies this definition and discusses the commitment as a motivator to stay with a supplier. Moreover, commitment is an essential dimension of relationship quality and is a powerful sign of the quality of the relationship [62]. Besides, [63], claims that commitment stabilizes relationships because the actors involved are ready to make certain efforts to preserve the relationship. In the B2B context, commitment is undoubtedly the most frequently studied variable [60, 64, 65]. At the level of the operational definition of commitment, [60] considers the importance of relationship to the respondent, and belief about working to maintain the relationship as dimensions to measure relationship commitment.

### 3.2. Antecedents of relationship quality

#### 3.2.1. Relational selling behavior

Behaviors of medical representatives are treated as a critical element in building long-term relationships. The way a medical representative reacts to different elements within the sale encounters with a specific physician can predict the future possibilities of the relationship [7, 27, 66]. Relational selling behavior is a multi-dimensional construct that refers to a “behavioral tendency exhibited by some sales representatives to their customers” [7] (p.71). [67] investigated the effect of marketing activities on relationship quality in the bank setting, and the findings suggested that greater employee’s relational selling behavior, client orientation, and better financial provision and employee’ attributes resulted in higher relationship quality. In the telecom sector, [68] also examined empirically the mediating role of relationship quality on the relationships between relational selling behavior, network quality, service recovery, and loyalty. The outcomes showed that relational selling behavior, network quality and service recovery indirectly influenced loyalty through the mediation of relationship quality consisting of satisfaction and trust.

Relationship selling behaviors emerged as a construct of three elements, namely, mutual disclosure, contact intensity, and cooperative intentions where these three-elements are a part of the relationship quality model that aims to show structural characteristics of long-term sales relationships in service selling [27, 69]. Concerning the first dimension of relational selling behavior, mutual disclosure refers to the willingness of the parties involved to reveal mutually critical personal or business information within a collaborative relationship [44, 70–72]. Successful relational sellers are more likely to be effective at soliciting customer disclosure of personal information and needs-related information and to be perceived by the customer as reciprocating in kind [7, 64]. Mutual disclosure as a dimension of relational selling behavior is considered as reciprocal concept, that is, the perception that another party is engaging in disclosure behavior toward oneself that is not being reciprocated often, which is read as a weakness on the other party’s part and may lead to an unhealthy relationship [70].

The contact intensity, on the other hand, refers to the frequency in which the medical representative communicates (face-to-face or indirectly) with the physician either for personal or business purposes. It reflects an effort on the part of the salesperson to keep the communication channels open with the customer and exhibit a commitment to the relationship [73]. Relational sellers appear to seek out their customers on a relatively frequent basis, through simply staying in touch, periodic needs reassessment and purchase reinforcement [7]. Parties in a relational context cannot be expected to trust each other in critical moments if these constitute their only opportunity to interact [27, 64, 74, 75].

Finally, the cooperative intentions refer to the positive attitude towards sharing information or problem solving within a collaborative relationship. These intentions, or goals, provide an active behavioral outcome rather than mere willingness [66]. Cooperative norms reflect the expectations that two exchanging parties have about working together to achieve mutual and individual goals jointly. In other words, cooperative norms do not imply one party’s acquiescence to another’s needs but rather that both parties behave in a manner that suggests they understand that they must work together to be successful [51, 69, 76, 77]. Based on these findings, this study assumes that:

**H1**. Relational selling behavior has a significant effect on relationship quality.

#### 3.2.2. Similarities

A crucial part of the antecedent variables of relationship quality relates to the similarities between the medical representative and physician. The general presumption here is that salespeople are more likely to be successful if they have some characteristics in common with their prospects [30, 78–80]. Generally, similarities can be measured using three dimensions, namely, appearance, lifestyle and socioeconomic status [7, 34]. Important evidence was taken from outside the marketing literature, for example, social psychology, counseling, communication, suggest that similarities among individuals in a relational context influence relationship satisfaction [22, 78, 81]. Additionally, [82] indicates that the similarity between the customer and service representative is considered a determinant of trust in a service provider. Likewise, [83] studied the effect of franchisee personality traits on relationship quality. Four out of the five personality traits were predicted to influence the relationship quality. The results showed that relationship quality was affected positively by dimensions of “agreeableness”, “conscientiousness” and “emotional stability”, while relationship quality was affected negatively by “extraversion”. Furthermore, [78, 84] argue that similarity in conscientiousness is associated with higher relationship quality. Moreover, internal similarity, such as personality was also found to influence several measures of relationship quality including trust [85]. On the other hand, [7] states that higher levels of perceived similarity improved short-term performance like higher levels of new sales, however, it does not improve the longer-term performance, namely, buyer perceptions of relationship quality. Based on the previous argument, this study explored the influence of similarity on the relationship quality.

**H2**. Similarities between medical representatives and physicians have a significant effect on relationship quality.

#### 3.2.3. Expertise

Expertise is defined by [48] as “the extent to which source possesses the knowledge, experience relevant to a particular topic” (p.41). Expertise has been described as a component of source characteristics in communication theory-based marketing research [23, 86, 87]. Generally, the salesperson that has a breadth of relevant information, which serves as a competent source, can be trusted [7]. Consequently, the customer’s perception of the expertise of salesperson product and market knowledge will enhance the relationship quality both parties have. [88] argues that expertise is more effective than referent power in establishing successful customer relationships. While [81, 82] argue that perceived salesperson expertise affects consumer’s purchase intention, [7] suggest that salesperson expertise leads to higher levels of relationship quality. Likewise, [89, 90] state that higher expertise is associated with higher trust. As a result, it can be assumed that expertise is considered as a variable that enhances the medical representative relationship quality with the physician, where physicians become satisfied with the salespeople because they possess a level of knowledge concerning their product class. This, in turn, helps physicians to provide quality treatment for their patients. Therefore, salesperson knowledge is considered as elements to measure salesperson expertise. Accordingly, the third hypothesis is formulated as:

**H3**. Expertise has a significant effect on relationship quality.

#### 3.2.4. Ethical behavior

Ethical behavior plays an important role in securing relationship quality [28, 29, 48, 91, 92]. The role of medical representatives in their interaction with physicians focuses on the dissemination of pharmaceutical product information by providing the physician with accurate information to assist physicians to prescribe accurate medication for their patients. Unethical medical representative behavior such as misrepresentation of warranties and overstating of their product capabilities could cause physician disputes and lower the perception of the quality in the relationship [91, 93]. Moreover, several conditions may lead the salesperson to frequently be confronted with opportunities to breakthrough corporate ethical policies. For instance, a medical representative works for long periods without supervision [28, 29, 48]. Furthermore, a salesperson may feel pressured to perform more when compared to other career types, hence, he may be forced to engage in unethical behavior to maintain his/her job [31, 93]. Ethical dissemination of information by medical representatives could also include refraining from exaggerating the product capabilities as well as informing the physician about possible side effects and complications [33, 94]. Although an ethical medical representative should be aware of a competitor’s strengths and weaknesses, they should not disparage competitors’ products [48]. As a result, the fourth hypothesis assumes that:

**H4**. Ethical medical representative behavior has a significant effect on relationship quality.

### 3.3. The anticipation of future interaction

The anticipation of future interaction is identified by [7] as “an outcome goal of dyadic encounters” (p.70). Low expectations of future exchange would be an outgrowth of current relational problems, whereas high expectations of future interchange would reflect a favorable perception of the current relationship. Hence, the best predictor of a physician’s likeability of seeking future interaction with a medical representative is the quality of the present relationship. [7, 95] proposes that relationship quality had a significant influence on the customer’s anticipation of future interaction with the salesperson.

Relationship quality serves as an indicator of the health and future wellbeing of long-term service sales relationships, thus, confronts the uncertainty that is often present in a business to business context [64]. Relationship quality contributes to a lasting bond by offering assurance that the medical representative will continue to meet the customer’s expectations (satisfaction), and not knowingly distort information or otherwise subvert the physician’s interests (trust) [91, 96]. The continuity of interaction that relationship quality provides an ongoing opportunity for pharmaceutical companies to identify the customers’ unmet needs and wants [97]. Also, it will facilitate future interaction and increase new business opportunities in the future [93]. Therefore, the current study assumes that:

**H5**. Relationship quality has a significant effect on the anticipation of future interaction.

**Fig 1:**
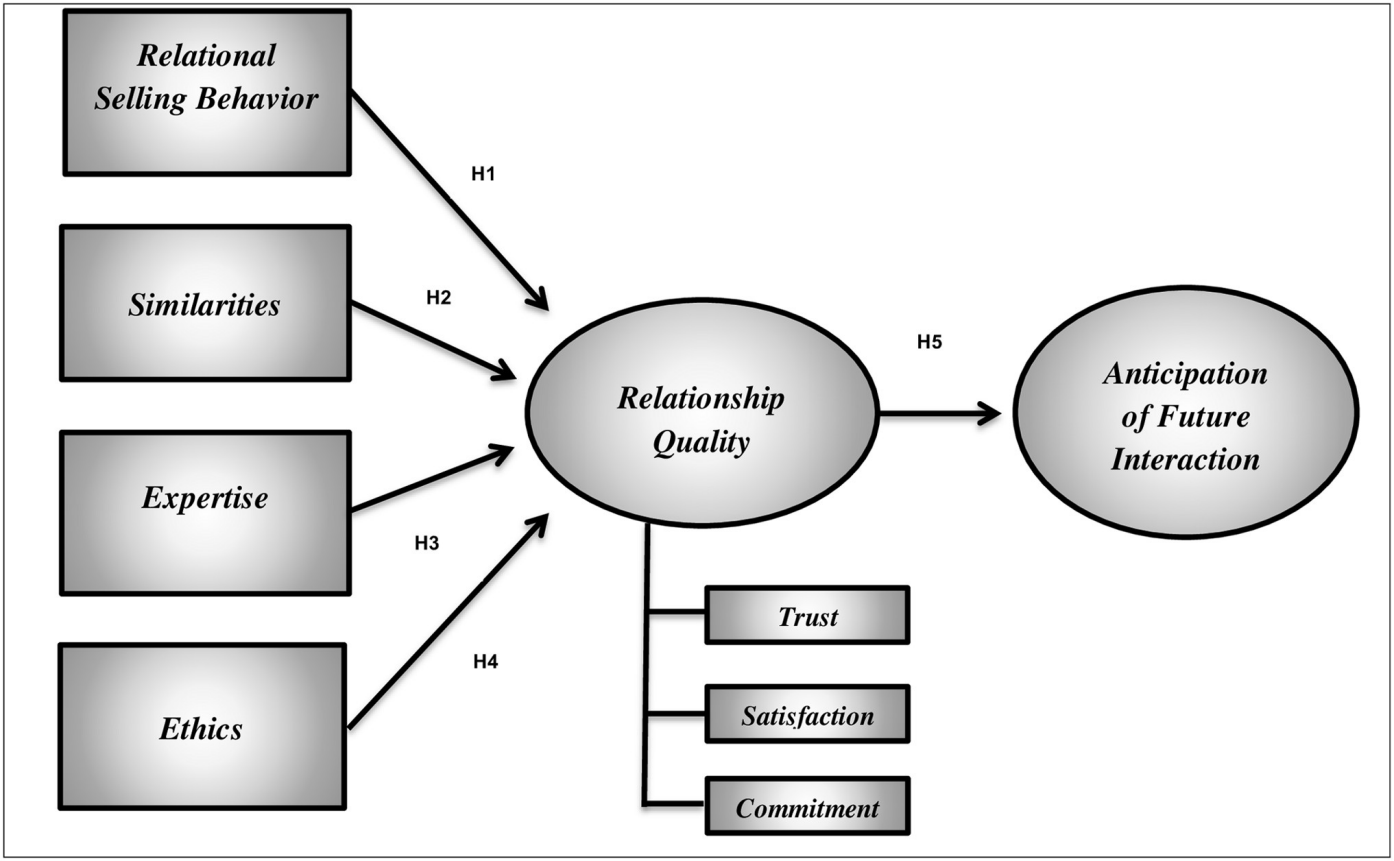
Research model of relationship quality [adopted from [7]].

The model used in this research is shown in Fig 1. The focal variable of this study is relationship quality between medical representatives and physicians as perceived by the physician. Also, this study narrows its concern to those settings in which relationship marketing is appropriate and the medical representative assumes the key implementation role.

## 4. Research methodology

To test for causal relationships between the variables of our model, a quantitative approach was adopted [98]. Consequently, a self-administrated questionnaire was used to collect data from the selected sample. Physicians working in the public healthcare sector in Jordan were selected as a population for this research. According to World Health Organization, the public sector includes the MOH, which includes tertiary hospitals, primary health care centers, and rural health posts, Royal Medical Services (RMS, the military sector), Jordan University Hospital (JUH), and King Abdullah University Hospital (KAUH). Jordan has implemented a network of public health care facilities, including 104 hospitals, 64 comprehensive health care centers, 377 primary health care centers, 238 secondary health care centers, 416 maternal child-health care centers and 285 dental clinics.

The reasons for choosing physicians in the public healthcare sector relate to the assumption that physicians are considered the key customers for pharmaceutical companies as they played a relevant role in the B2B buying process as initiators, deciders and users for the pharmaceutical product [8]. Consequently, a purposive sampling method was employed to select a sample of (N = 374) out of 500 distributed questionnaires with a 74.8% response rate. 126 questionnaires were not used in the analysis while they represented the non-returned questionnaires. Moreover, the sample consisted of physicians working at Al Basheer Hospital, Prince Hamza Hospital, Jordanian Royal Medical Services, Jordan University Hospital, and King Abdullah University Hospital. However, consents from participants were not obtained as the data were analyzed anonymously. The needed data has been collected from February 2019 to May 2019.

Concerning the measures used, Table 1 shows the origin of the study measures and the number of items for each scale. The scales were modified to reflect the relevant characteristics of the pharmaceutical context.

**Table 1:** Measures utilized.

Variables /Dimensions	Resource	Number of items
• Relationship quality (trust, satisfaction and commitment)	[7, 60, 67, 85, 99]	15
• Similarities	[85]	3
• Expertise	[60, 100]	5
• Relational selling behavior	[7, 60, 67, 100]	11
• Ethics	[48]	5
• The anticipation of future interaction	[99]	5

## 5. Results

As seen in Table 2, the Cronbach’s Alpha value for each variable was above 0.60 and as a result, the reliability analysis indicates the internal consistency reliability of all the scales items [98].

**Table 2:** Cronbach’s alpha for each construct.

Variables	Cronbach’s Alpha for items before refinement		Cronbach’s Alpha for items after refinement	
	Number of items	Cronbach’s Alpha	Number of items	Cronbach’s Alpha
*Relationship quality*	15	0.899	13	0.952
*Similarities*	3	0.552	2	0.659
*Expertise*	5	0.888	5	0.888
*Relational behavior*	11	0.866	9	0.897
*Ethics*	5	0.877	5	0.877
*Anticipation of future interaction*	5	0.931	5	0.931

A Confirmatory Factor Analysis (CFA) was also employed to assess the construct validity of the study instrument as seen in Table 3. Factor loadings with more than 0.40 were accepted as recommended by [101]. According to components matrix analysis outputs, it can be noticed that the factor loadings for all items were above 0.4 except for two items (T6, C2, SimG, CI2, M1) (these were more than two items), which all had a value less than 0.4, therefore, they were excluded. Moreover, no issues were found regarding the composite reliability (CR) and average variance extracted (AVE) as the values for all of the items were above 0.70 and 0.50 respectively [101].

**Table 3:** CFA results.

Constructs and items		EFA results		CFA results		
***Relationship Quality (Trust)***		***Factor Loadings***	***Eigen Value***	***Factor Loadings***	***Composite reliability***	***Average variance extracted***
T1	I feel that this medical representative is trustworthy.	0.841	3.799	0.80	60.2%	95.2%
T2	I believe that this medical representative can keep his/her promises.	0.869		0.82		
T3	I believe that this medical representative is honest.	0.834		0.77		
T4	I feel that this medical representative is sincere.	0.873		0.76		
T5	I think that this medical representative is reliable.	0.865		0.83		
T6	I think that this medical representative would not tell a lie even if he/she could gain by it.	Deleted				
***Relationship Quality (Satisfaction)***						
S1	I am satisfied with this medical representative.	0.811	3.553	0.74		
S2	I am pleased with this medical representative.	0.821		0.81		
S3	I think considering the medical representative is favorable.	0.691		0.72		
S4	I feel contentment for this medical representative.	0.778		0.77		
S5	I have a healthy relationship with a considered medical representative.	0.752		0.80		
***Relationship Quality (Commitment)***						
C1	I believe we are mutually committed to this relationship.	0.874	2.524	0.79		
C2	I think that this medical representative is prepared to make short-term sacrifices to maintain our relationship.	Deleted				
C3	I believe this medical representative views our relationship as a long-term partnership.	0.905		0.74		
C4	I believe this medical representative will do his/her maximum effort to maintain our relationship.	0.915		0.73		
***Similarities***		***Factor Loadings***	***Eigen Value***	***Factor Loadings***	***Composite reliability***	***Average variance extracted***
**Sim 1**	Please assess the similarity in personality between you and the medical representative you have considered.	0.802	1.616	0.62	55.4%	70.7%
**Sim 2**	Please assess the similarity in appearance between you and the medical representative you have considered.	0.836		0.85		
**Sim G**	Please assess the similarities in gender between medical representatives and physicians.	0.523		Deleted		
***Expertise***		***Factor Loadings***	***Eigen Value***	***Factor Loadings***	***Composite reliability***	***Average variance extracted***
**Ex1**	I think that this medical representative is very knowledgeable.	0.592	3.564	0.47	51.7%	87.4%
**Ex2**	I think that this medical representative knows his/her product very well.	0.905		0.93		
**Ex3**	I think this medical representative is an expert.	0.918		0.91		
**Ex4**	I think that this medical representative is an excellent source of accurate product information.	0.868		0.85		
**Ex5**	I think that this medical representative knows a lot about his/her product.	0.893		0.84		
***Relational Behavior (Contact Intensity)***		***Factor Loadings***	***Eigen Value***	***Factor Loadings***	***Composite reliability***	***Average variance extracted***
**CI 1**	I was contacted by a medical representative who wanted to stay "in touch”.	0.756	2.320	0.68	50.9%	89.3%
**CI 2**	This medical representative frequently visits me.	0.458		Deleted		
**CI3**	I think this medical representative is efficient in taking my needs.	0.882		0.64		
**CI4**	I think that this medical representative knows my needs and wants well.	0.872		0.70		
***Relational Behavior (Mutual Disclosure)***						
**M1**	I have confidence in the considered medical representative private information about me (e.g. income).	0.594	2.175	Deleted		
**M2**	I have expressed to considered medical representative my liking for him/her as a person	0.705		0.64		
**M3**	I have told the medical representative about the diagnosis and treatment mistakes I’ve made in the past.	0.770		0.81		
**M4**	I have expressed to the medical representative about my dissatisfaction with other medical representatives.	0.855		0.72		
***Relational Behavior (Cooperative intention)***						
**Coo1**	I believe this medical representative has expressed a desire to develop a long-term relationship.	0.830	2.241	0.82		
**Coo2**	I believe this medical representative has expressed a willingness to help.	0.917		0.70		
**Coo3**	I believe that this medical representative will help me to obtain update researches about his/her company products.	0.843		0.69		
***Ethics***		***Factor Loadings***	***Eigen Value***	***Factor Loadings***	***Composite reliability***	***Average variance extracted***
**E1**	I believe that this medical representative never lies about the competition to make sales.	0.839	3.383	0.81	57.9%	87.0%
**E2**	I think that this medical representative would not exaggerate the features and benefits of his/her products.	0.853		0.86		
**E3**	I think that this medical representative would not pass the blame for something he/she did wrong to someone else.	0.835		0.82		
**E4**	I believe that this medical representative cares about the patient’s interest.	0.832		0.70		
**E5**	I believe that this medical representative will never sell dangerous products.	0.749		0.58		
***The anticipation of Future Interaction***		***Factor Loadings***	***Eigen Value***	***Factor Loadings***	***Composite reliability***	***Average variance extracted***
**Anti1**	I am pleased to repeat prescribing considered medical representative’s product.	0.897	4.021	0.84	74.5%	93.6%
**Anti2**	Next time when needed, I will prescribe his/her product.	0.925		0.93		
**Anti3**	I do not mind prescribing other products for this medical representative.	0.892		0.87		
**Anti4**	I do not mind re-prescribing his/her product again.	0.900		0.88		
**Anti5**	I will recommend his/her product to my colleagues’ physicians.	0.870		0.79		

Structural Equation Modeling (SEM) was employed to test the research hypotheses. The research model described in Fig 1 was estimated using AMOS 22 with the maximum likelihood estimation method. The Model fit indices in Table 4 illustrate that the research model fits the data well. Scaled X2/df = 2.471, SRMR = 0.0564, GFI = 0.902, NFI = 0.903, CFI = 0.939, RMSEA = 0.063. According to [102, 103], the appropriate scores for the indices should be along (P ≥ 0.05) for the Chi-square, goodness-of-fit index (GFI ≥ 0.90), adjusted goodness-of-fit index (AGFI ≥ 0.80), comparative fit index (CFI ≥ 0.90), normed fit index (NFI ≥ 0.90), standardized root-mean-square residual (SRMR ≤ 0.08) and root mean square error of approximation (RMSEA < 0.10).

**Table 4:** Measurement model fit indices.

Model	X^2^	Df	P	X^2^/df	SRMR	NFI	CFI	GFI	RMSEA
Research Model	1373.836	556	0.000	2.471	0.0564	0.903	0.939	0.902	0.063

Furthermore, the results of the hypothesis testing are presented in Table 5. The paths for (H1, H3, H4, and H5) in the research model were supported, with only H2 rejected owing to the significance value being above 0.005 [104, 105].

**Table 5:** Results of hypotheses testing using AMOS 22.

*Research Proposed Paths*	*Estimate*	*S*.*E*.	*t-value*	*p-value*	*Hypothesis supported*
Relational selling behavior → Relationship quality	0.230	0.086	2.684	0.007	Yes
Similarities → Relationship quality	-0.001	0.006	-0.160	0.873	No
Expertise → Relationship quality	0.339	0.049	6.952	0.000	Yes
Ethics → Relationship quality	0.505	0.104	4.865	0.000	Yes
Relationship quality → Anticipation of future interaction	1.249	0.089	14.006	0.000	Yes

## 6. Discussion and conclusion

Using a sample of (N = 374) physicians in Jordan, this study aimed to investigate the antecedents and consequences of relationship quality in the pharmaceutical Industry. Aside from the similarities’ influence on relationship quality, all the proposed hypotheses generated by this study were supported, so that the developed model was tested and validated. Generally, this study sheds some light on the importance of developing and improving a quality relationship between physicians in the public healthcare sector, on the one side, and the medical representative of pharmaceutical companies, on the other. Consequently, this study contributes to the literature by filling the gap that exists in linking the antecedents of relationship quality and relationship quality with the anticipation of future interaction in the Jordanian pharmaceutical industry.

Regarding the antecedents of relationship quality, the results showed that relationship quality was affected positively by relational selling behaviors, which implied that relational selling behaviors were considered an effective factor for maintaining a good relationship quality. This finding is supported by previous findings in the literature [7, 51, 67, 68, 70, 72], which similarly found a positive link between these two factors. Consequently, pharmaceutical firms with medical representatives who engage in selling behaviors should focus on long-term relationship aspects, namely, mutual disclosure, high contact intensity, and cooperative intentions. This will ensure that they enjoy the benefits of favorable relationship quality, which, in turn, will reflect positively on future interaction. It can be also concluded that relational selling behaviors performed by medical representatives should focus on the development of mutual-benefit relationships (i.e. win-win situations). Particularly, stronger relationships allow for greater trust, satisfaction, and commitment, thereby leading to lower customer turnover [43]. Moreover, this finding also means that customers are more open to problem-solving initiatives when relational selling behaviors are performed by the medical representative. Such findings provided an implication for medical representatives to maintain regular contact and contracts with their customers to encourage physicians to disclose information about themselves. Furthermore, medical representatives should disclose their intentions to cooperate to maintain a high relationship quality with physicians.

Regarding the relationship between similarities and relationship quality, the results of this study revealed that there were no significant relationships between these variables. This could indicate that similarities between medical representatives and physicians were not a predictor of the quality of long-term relationships in this context. This result fosters the previous findings in the literature [7] while this result contrast some previous findings (e.g. [78, 81, 83, 84]). Particularly, the personality, appearance and gender similarities are irrelevant to establish a relationship quality. This could be attributed to the perspective of physicians in constructing the relationship with a medical representative where physicians tend to build this relationship based on a scientific and a rational basis instead of a discrimination and bias basis. For example, the effect of salesperson’s listening behavior and empathy on the relationship quality was studied by [106] with customers in the banking sector context. To add more, non-inclusion of gender similarity as a predictor of relationship quality might indicate that physicians within this sample did not distinguish between males and females in this field, and considered the male and female performances equally, not male over female or female over male. Such arguments might be attributed to the level of gender equality in Jordan where males and females in the Jordanian environment have equal access to good-quality education, equal rights, and opportunities.

Furthermore, the findings of this study documented that relationship quality was also affected positively by the medical representative’s expertise, which indicated that physicians within the sample took into account the medical representatives’ expertise level when considering the relationship quality. Furthermore, pharmaceutical firms with a high level of medical representative expertise should enjoy the benefits of high relationship quality, which would reflect positively on future interactions. These results might be attributed to the essential role of knowledge and expertise in the healthcare sector, and the importance of accuracy in this field as well as the consequences of inaccuracy [8]. Thus, it could be argued that expertise was considered an important factor in building trust where accurate information provided by the medical representatives was anticipated to help the physician in choosing the appropriate medication for his/her patient. Accordingly, it was expected that a physician would be attracted to spend the time and effort required to develop a business long-term relationship with a medical representative who was an expert in his/her product and field. Such an outcome provides an implication for medical representatives to show some degree of expertise to have an opportunity to form a good relationship quality, especially in the assessment process that the physician has for the medical representative. Therefore, it was expected that the medical representative knowledge might be critical to the first impression that a physician made about the medical representative with whom he/she dealt.

This issue was confirmed by [107] who studied the effect of knowledge management and global mindset on client-vendor relationship quality. The survey was conducted on software and services companies in India. The results showed that the quality of the client-vendor relationship was positively and significantly affected by both knowledge management and a global mindset. Also, [108] tested the mediating roles of cultural sensitivity and information exchange in the impact of antecedent factors and market orientation on relationship quality in Vietnamese exporter’s field. The outcomes revealed that market orientation had both direct and indirect effects, mediated by cultural sensitivity and information exchange, on relationship quality. Thus, medical representatives should improve their knowledge about their products to build a healthy long-term quality relationship with physicians.

Regarding the ethical dimension, the result of this study also pointed out that the quality of the relationship was affected positively by the medical representatives’ ethical behavior. This finding suggested that the ethics of the medical representative was a predictor of relationship quality as previously demonstrated by the literature. Besides, pharmaceutical companies with a high level of medical representative ethical behavior enjoyed the benefits of favorable relationship quality, which reflected positively the future interaction when compared to pharmaceutical companies with a low level of medical representative ethical behavior [48]. This finding could be attributed also to the nature of the healthcare sector, the essential role of ethics in this field and the consequences of any unethical behavior in this sector [14, 109].

Accordingly, it could be argued that physicians should take into consideration the level of ethics in determining their relationships with medical representatives. For example, the ethical level of medical representatives would be determined by factors such as never lying about the competitor to make sales, not exaggerating the features and benefits of their products, not passing the blame for something they had done wrong to someone else, caring about patient interests and never selling dangerous products. These ethical attributes would result in good relationship quality with physicians and thus lead to long-term business relationships [110]. This outcome provides an implication for medical representatives to engage in a high level of ethical behavior and to be aware of the complications of any unethical or corrupt business practices.

Finally, similar to previous studies, for example, [7, 95, 111], the results of the study also revealed that anticipation of future interaction was influenced positively by relationship quality, which implied that the relationship quality between a physician and medical representative served as an indicator for a healthy and a long-term sales relationships. Furthermore, maintaining the quality of the relationship would facilitate future interaction and increase future business opportunities. This suggested that pharmaceutical firms with a high relationship quality between medical representatives and physicians enjoyed high future interactions. However, pharmaceutical firms with low relationship quality between medical representatives and physicians suffered from low future interactions. Accordingly, it was expected that low future interaction is considered an outgrowth of current relational problems, whereas high future interaction was considered a reflection of a favorable perception of the current relationship. Consequently, it is recommended to pharmaceutical companies in Jordan to focus on encouraging medical representatives’ ethical behavior, expertise and relational selling behavior owing to the importance of these factors in improving the relationship quality with physicians, which will reflect positively on future interaction. Most importantly, conflict handling among mutual relationship partners is essential and affects relationship quality. This issue has been confirmed by [112] who examined the effect of relational dynamics, namely, trust, personalization, communication, conflict handling, and empathy, on relationship quality, in Malaysia and New Zealand. The results showed that communication, trust, and empathy were significantly related to relationship quality in both countries, whereas relationship quality was significantly affected by personalization in New Zealand but not in Malaysia. The results also revealed that conflict handling was significantly and marginally associated with relationship quality in New Zealand and Malaysia, respectively.

## 7. Implications, limitations and further research

Based on the finding of this research, various managerial implications can be derived and used by sales and marketing managers in pharmaceutical companies in Jordan to increase relationship quality with their customers. Thus, managers should develop services marketing strategies that deepen and enhance long term customer relationships, meantime they can secure a sustainable competitive advantage that will enhance the opportunities for future business and facilitate interaction in the future. Also, managers can utilize the connection between relational selling behaviors (contact intensity, mutual disclosure, and cooperative intentions) and relationship quality (trust, satisfaction, and commitment). When hiring a medical representative, marketing and sales managers can screen for the social skills that facilitate establishing a quality relationship, which leads to maintaining long-term interpersonal relationships. This screening can be done via role-plays within the interview. Furthermore, managers could use the positive effect of ethical behavior on the quality of relationships through training programs that focus on learning the impact of ethics on relationships, draw attention to potential ethical issues, and greater awareness of the complications that follow unethical behaviors. Finally, owing to the impact of expertise on relationship quality, managers should focus on training courses and continuous learning programs for the medical representatives, especially the latest updates and pharmaceutical product studies. These studies would also reflect positively on relationship quality.

For theoretical implications, the general purpose of this investigation for researchers was to explore the antecedents of relationship quality as well the effect of relationship quality on the future interaction for the pharmaceutical context, from the customer perspective (i.e. physicians’ perspective). Using a quantitative research method, self- administrated questionnaire, and SEM analysis technique to test the hypotheses, the overall results provide strong support for the relationship quality model (Fig 1) which includes the relationship quality as an intermediating variable as well as the antecedents’ variables (expertise, relational selling behavior, and relational ethics) and consequence of relationship quality (anticipation of future interaction). However, the similarities between medical representative and their customers found to have no significant effect on the relationship quality. Hence, as more than one study could enhance the construct validity. In addition to the shortage of studies applied the relationship quality model in the pharmaceutical firms, the present study offered a piece of evidence that strengthens the validity and the applicability of the relationship quality model in the pharmaceutical context.

As with all social science studies, some limitations exist that could provide fruitful avenues for future research. First, the research population was compromised to the physicians in the public healthcare sector and thus physicians from private healthcare sector were not included, due to the differences in buying process of these businesses, future studies could examine the study model on physicians from the private healthcare sector, also, future studies could consider the perspectives of medical representatives or both physician and medical representatives. Second, while this study examines the impact of relationship quality on the anticipation of future interaction, future research could examine the influence of relationship quality on the actual retention rates. Finally, considering that the majority of studies examine pharmaceutical marketing issues in the traditional brick and mortar context, future research could employ this model in the online context and examine the influence of social media on the work of pharmaceutical firms.

## Supporting information

S1 Data (SAV)

S1 File (DOCX)
