# Supplementary material for: Antecedents and consequences of relationship quality in pharmaceutical industries: A structural equation modelling approach
Source: PLoS One. 2023 Jan 20;18(1):e0279824. doi: 10.1371/journal.pone.0279824 (PMC9858337; doi:10.1371/journal.pone.0279824)
Supplement: S1 File — (DOCX) [file pone.0279824.s002.docx]

**Antecedents and Consequences of Relationship Quality in Pharmaceutical Industries: a structural equation modelling approach**

**Supplementary file 1 - Common method bias:**

To examine the common method bias, the single factor Harman’s test (1976) was employed. Consequently, using SPSS confirmatory factor analysis the test was conducted by loading all the 44 items of the study into one factor. Accordingly, the results show that the first factor emerging accounted for 45.660% of variance, therefore indicating that common method bias is not a major concern in this research since this percentage is less than Podsakoff’s *et al.* (2003) 50% threshold.

References

Harman, H.H. (1976), Modern Factor Analysis, University of Chicago Press, Chicago, IL.

Podsakoff, P.M., MacKenzie, S.B., Lee, J.Y. and Podsakoff, N.P. (2003), “Common method biases in

behavioral research: a critical review of the literature and recommended remedies”, Journal of

Applied Psychology, Vol. 88 No. 5, pp. 879-903.

GET

FILE='C:\Users\LENOVO\Downloads\DATA.sav'.

Warning # 5281. Command name: GET FILE

SPSS Statistics is running in Unicode encoding mode. This file is encoded in

a locale-specific (code page) encoding. The defined width of any string

variables are automatically tripled in order to avoid possible data loss. You

can use ALTER TYPE to set the width of string variables to the width of the

longest observed value for each string variable.

DATASET NAME DataSet1 WINDOW=FRONT.

FACTOR

/VARIABLES T1 T2 T3 T4 T5 T6 St1 St2 St3 St4 St5 Cm1 Cm2 Cm3 Cm4 Si1 Si2 Si3 Ex1 Ex2 EX3 Ex4 Ex5

Ci1 Ci2 Ci3 Ci4 Md1 Md2 Md3 Md4 Co1 Co2 Co3 Et1 Et2 Et3 Et4 Et5 An1 An2 An3 An4 An5

/MISSING LISTWISE

/ANALYSIS T1 T2 T3 T4 T5 T6 St1 St2 St3 St4 St5 Cm1 Cm2 Cm3 Cm4 Si1 Si2 Si3 Ex1 Ex2 EX3 Ex4 Ex5

Ci1 Ci2 Ci3 Ci4 Md1 Md2 Md3 Md4 Co1 Co2 Co3 Et1 Et2 Et3 Et4 Et5 An1 An2 An3 An4 An5

/PRINT INITIAL EXTRACTION

/CRITERIA FACTORS(1) ITERATE(25)

/EXTRACTION PC

/ROTATION NOROTATE

/METHOD=CORRELATION.

**Factor Analysis**

[DataSet1] C:\Users\LENOVO\Downloads\DATA.sav

| **Communalities** | | |
| --- | --- | --- |
|  | Initial | Extraction |
| T1 | 1.000 | .513 |
| T2 | 1.000 | .606 |
| T3 | 1.000 | .525 |
| T4 | 1.000 | .517 |
| T5 | 1.000 | .594 |
| T6 | 1.000 | .107 |
| St1 | 1.000 | .621 |
| St2 | 1.000 | .575 |
| St3 | 1.000 | .501 |
| St4 | 1.000 | .586 |
| St5 | 1.000 | .562 |
| Cm1 | 1.000 | .572 |
| Cm2 | 1.000 | .102 |
| Cm3 | 1.000 | .574 |
| Cm4 | 1.000 | .565 |
| Si1 | 1.000 | .004 |
| Si2 | 1.000 | .002 |
| Si3 | 1.000 | .022 |
| Ex1 | 1.000 | .242 |
| Ex2 | 1.000 | .570 |
| EX3 | 1.000 | .618 |
| Ex4 | 1.000 | .610 |
| Ex5 | 1.000 | .576 |
| Ci1 | 1.000 | .414 |
| Ci2 | 1.000 | .008 |
| Ci3 | 1.000 | .435 |
| Ci4 | 1.000 | .492 |
| Md1 | 1.000 | .036 |
| Md2 | 1.000 | .318 |
| Md3 | 1.000 | .430 |
| Md4 | 1.000 | .344 |
| Co1 | 1.000 | .570 |
| Co2 | 1.000 | .503 |
| Co3 | 1.000 | .473 |
| Et1 | 1.000 | .542 |
| Et2 | 1.000 | .497 |
| Et3 | 1.000 | .552 |
| Et4 | 1.000 | .542 |
| Et5 | 1.000 | .509 |
| An1 | 1.000 | .648 |
| An2 | 1.000 | .667 |
| An3 | 1.000 | .662 |
| An4 | 1.000 | .657 |
| An5 | 1.000 | .630 |
| Extraction Method: Principal Component Analysis. | | |

| **Total Variance Explained** | | | | | | |
| --- | --- | --- | --- | --- | --- | --- |
| Component | Initial Eigenvalues | | | Extraction Sums of Squared Loadings | | |
|  | Total | % of Variance | Cumulative % | Total | % of Variance | Cumulative % |
| 1 | 20.091 | 45.660 | 45.660 | 20.091 | 45.660 | 45.660 |
| 2 | 2.217 | 5.038 | 50.698 |  |  |  |
| 3 | 2.084 | 4.737 | 55.435 |  |  |  |
| 4 | 1.723 | 3.915 | 59.350 |  |  |  |
| 5 | 1.618 | 3.676 | 63.027 |  |  |  |
| 6 | 1.378 | 3.131 | 66.157 |  |  |  |
| 7 | 1.123 | 2.552 | 68.710 |  |  |  |
| 8 | .937 | 2.129 | 70.838 |  |  |  |
| 9 | .923 | 2.098 | 72.936 |  |  |  |
| 10 | .835 | 1.898 | 74.834 |  |  |  |
| 11 | .780 | 1.773 | 76.607 |  |  |  |
| 12 | .758 | 1.722 | 78.329 |  |  |  |
| 13 | .739 | 1.679 | 80.008 |  |  |  |
| 14 | .681 | 1.548 | 81.556 |  |  |  |
| 15 | .605 | 1.375 | 82.931 |  |  |  |
| 16 | .575 | 1.307 | 84.238 |  |  |  |
| 17 | .541 | 1.229 | 85.467 |  |  |  |
| 18 | .512 | 1.164 | 86.631 |  |  |  |
| 19 | .491 | 1.116 | 87.746 |  |  |  |
| 20 | .444 | 1.010 | 88.756 |  |  |  |
| 21 | .433 | .983 | 89.740 |  |  |  |
| 22 | .391 | .890 | 90.629 |  |  |  |
| 23 | .357 | .812 | 91.441 |  |  |  |
| 24 | .337 | .766 | 92.207 |  |  |  |
| 25 | .326 | .742 | 92.949 |  |  |  |
| 26 | .298 | .677 | 93.626 |  |  |  |
| 27 | .287 | .651 | 94.277 |  |  |  |
| 28 | .283 | .642 | 94.920 |  |  |  |
| 29 | .241 | .549 | 95.468 |  |  |  |
| 30 | .217 | .493 | 95.961 |  |  |  |
| 31 | .203 | .460 | 96.421 |  |  |  |
| 32 | .194 | .442 | 96.863 |  |  |  |
| 33 | .181 | .412 | 97.276 |  |  |  |
| 34 | .169 | .385 | 97.661 |  |  |  |
| 35 | .149 | .339 | 97.999 |  |  |  |
| 36 | .137 | .311 | 98.310 |  |  |  |
| 37 | .125 | .284 | 98.594 |  |  |  |
| 38 | .121 | .276 | 98.870 |  |  |  |
| 39 | .113 | .257 | 99.127 |  |  |  |
| 40 | .090 | .204 | 99.331 |  |  |  |
| 41 | .086 | .196 | 99.527 |  |  |  |
| 42 | .078 | .178 | 99.706 |  |  |  |
| 43 | .072 | .164 | 99.870 |  |  |  |
| 44 | .057 | .130 | 100.000 |  |  |  |
| Extraction Method: Principal Component Analysis. | | | | | | |

| **Component Matrix^a^** | |
| --- | --- |
|  | Component |
|  | 1 |
| T1 | .716 |
| T2 | .778 |
| T3 | .724 |
| T4 | .719 |
| T5 | .770 |
| T6 | -.327 |
| St1 | .788 |
| St2 | .758 |
| St3 | .708 |
| St4 | .766 |
| St5 | .749 |
| Cm1 | .756 |
| Cm2 | -.319 |
| Cm3 | .757 |
| Cm4 | .752 |
| Si1 | -.060 |
| Si2 | .046 |
| Si3 | -.147 |
| Ex1 | .492 |
| Ex2 | .755 |
| EX3 | .786 |
| Ex4 | .781 |
| Ex5 | .759 |
| Ci1 | .644 |
| Ci2 | .088 |
| Ci3 | .660 |
| Ci4 | .702 |
| Md1 | .189 |
| Md2 | .563 |
| Md3 | .656 |
| Md4 | .586 |
| Co1 | .755 |
| Co2 | .709 |
| Co3 | .688 |
| Et1 | .736 |
| Et2 | .705 |
| Et3 | .743 |
| Et4 | .736 |
| Et5 | .714 |
| An1 | .805 |
| An2 | .817 |
| An3 | .814 |
| An4 | .811 |
| An5 | .793 |
| Extraction Method: Principal Component Analysis. | |
| a. 1 components extracted. | |

**Supplementary file 2 - Questionnaire Development**

The research questionnaire consists of two parts; the first one is general information about the participant (i.e. Physicians) namely: age, gender, job description, work place. In addition, some information about medical representative they select, namely: gender, relationship length. The second part includes statements that ask about respondent’s opinion, using Likert- scale, regarding relationship quality (trust, satisfaction, commitment), similarities, expertise, relational selling behavior (contact intensity, mutual disclosure, and cooperative intention), medical representative’s ethical behavior, and anticipation of future interaction.

Particularly, the items for each variable were taken from different sources as depicted in the supplementary table below. Where relationship quality consisted of three dimensions with total of (15 items), trust (6 items), satisfaction (5 items), commitment (4 items), similarities (3 items), expertise (5 items), relational selling behavior with a total of (11 items), contact intensity (4 items), mutual disclosure (4 items), cooperative intention (3 items), ethics (5 items), anticipation of future interaction (5 items).

***Items for Each Variable and Dimension***

| **Variable** | **Items** | **Source** | **Modification** |
| --- | --- | --- | --- |
| **Relationship Quality *(trust)*** | - My agent is trustworthy. | Crosby et al. (1990) | Modified |
|  | - My agent can be relied upon to keep his/her promises. | Crosby et al. (1990) | Modified |
|  | - Medical representative is honest | Alak (2010) | Modified |
|  | - A bank employee is sincere | Alak and Alnawas, (2010) | Modified |
|  | - A bank employee is reliable | Alak and Alnawas, (2010) | Modified |
|  | - The company, which produces the make of this car,  would not tell a lie, even if it could gain by it. | Kennedya et al. (2001) | Modified |

| **Relationship Quality *(Satisfaction)*** | - I am satisfied with medical representative | Crosby et al. (1990) | Modified |
| --- | --- | --- | --- |
|  | - I am pleased with a medical representative | Crosby et al. (1990) | Modified |
|  | - I think this medical representative is favorable | Crosby et al. (1990) | Modified |
|  | - ``Contented'' to ``Disgusted'' Seven scale point | Kennedya et al. (2001) | Modified |
|  | - I have a healthy relationship with considered medical representative. | New | New |
| **Relationship Quality *(Commitment)*** | - I believe we are both committed to this relationship. | Smith (1998) | Modified |
|  | - This rep is prepared to make short-term sacrifices to maintain our relationship. | Smith (1998) | Modified |
|  | - I believe we both view our relationship as a long-term partnership. | Smith (1998) | Modified |
|  | - I believe this medical representative will do his/ her maximum effort to maintain our relationship | Morgan and Hunt (1994) | Modified |
| **Similarities** | - Please assess the similarity between you and the sales rep you have considered on personality. (1 similar … 6 dissimilar) | Smith (1998) | Modified |
|  | - Please assess the similarity between you and the sales rep you have considered on appearance. (1 very similar … 4very dissimilar) | Smith (1998) | Modified |
|  | - Gender similarities or difference. | Smith (1998) | Modified |
| **Expertise** | - This salesperson is very knowledgeable. | Morgan and Hunt (1994) | Modified |
|  | - This salesperson knows his/her product line very well. | Morgan and Hunt, (1994) | Modified |

|  | - This salesperson is not an expert. (R) | Morgan and  Hunt, (1994) | Modified |
| --- | --- | --- | --- |
|  | - The salesperson was an excellent source of accurate  product information. | Kennedya et al.  (2001) | Modified |
|  | - This salesperson knew as much as he/she should have about the product. | Kennedya et al. (2001) | Modified |
| **Relational Selling Behavior. *(Contact Intensity)*** | - Was contacted by salesperson who wanted to stay "in  touch" and make sure I was still satisfied. | Crosby et al.  (1990) | Modified |
|  | - This salesperson frequently visits our place of business. | Morgan and  hunt (1994) | Modified |
|  | - This salesperson takes a lot of time learning our needs. | Morgan and  hunt (1994) | Modified |
|  | - A hotel employee knows guests’ needs and wants well. | Kim and  Cha (2002) | Modified |
| **Relational Selling Behavior. *(Mutual Disclosure)*** | - I have confided in the salesperson a lot of information  about my current financial situation (e.g., income, investments, and obligations). | Crosby et al. (1990) | Modified |
|  | - I have expressed to salesperson my liking and respect for him/her self as a person. | Crosby et al. (1990) | Modified |
|  | - Speak out about diagnosis and treatment mistakes. | New | New |
|  | - I have expressed to bank employee dissatisfaction with service. | Alak and Alnawas (2010) | Modified |
| **Relational Selling Behavior. *(Cooperative intension)*** | - My agent has expressed a desire to develop a long-term  relationship. | Crosby et al.  (1990) | Modified |
|  | - My agent has expressed a desire to develop a long-term  relationship. | Crosby et al.  (1990) | Modified |
|  | - Help in obtaining updated researches. | New | New |
| **Ethics** | - Lies about the competition in order to make sales. | Lagace et al. (1991) | Modified |
|  | - Exaggerates the features and benefits of his/her product. | Lagace et al. (1991) | Modified |
|  | - Passes the blame for something he/she did wrong to someone else. | Lagace et al. (1991) | Modified |
|  | - Is only interested on his own interest, not the clients’. | Lagace et al. (1991) | Modified |
|  | - Selling dangerous and hazardous products. | Lagace et al. (1991) | Modified |
| **Anticipation Of Future Interaction** | - I am pleasured to repeat prescribing considered medical representative’s product. | New | New |
|  | - The next time I buy a car, I will choose the same make  of car again. ``Unlikely'' to ``Likely''. | Kennedya et al.  (2001) | Modified |
|  | - I don’t mind prescribing other product for this medical representative. | New | New |
|  | - I don’t mind to re-prescribe his/her product again. | New | New |
|  | - I will recommend his/her product to my colleagues’ physicians. | New | New |
